# The Effect of Self-Management Training on Anxiety and Comfort of Burn Patients Candidate for Skin Grafting

**DOI:** 10.29252/wjps.9.2.200

**Published:** 2020-05

**Authors:** Zahra Shahryari, Mahnaz Seyedoshohadaee, Frough Rafii, Alice Khachian, Mokhtar Mahmoudi

**Affiliations:** Department of Med ical Surgical Nursing, School of Nursing and Midwifery, Iran University of Medical Sciences, Tehran, Iran

**Keywords:** Burn, Self-management training, Anxiety, Comfort, Skin graft

## Abstract

**BACKGROUND:**

Self-management programs on needs of burn patients are still essential. So this study determined the effect of self-management training on anxiety and comfort of burn patients who were candidate for skin grafting.

**METHODS:**

In a continuous sampling method in Shahid Motahari Burn Center affiliated to Iran University of Medical Sciences, 80 burn patients candidate for skin grafting were divided into equal groups of intervention and control. Educational intervention was undertaken in the form of booklet during two sessions before and after skin grafting. Visual comfort scale questionnaire and Spiel Berger state-trait anxiety inventory were completed by patients before training and one month after intervention.

**RESULTS:**

There was a significant difference between the frequency of comfort level in both groups before and after the intervention. The level of comfort in the intervention group increased more than control group. The mean level of patients’ anxiety showed a significant difference between groups and scores in intervention group were significantly more than control group.

**CONCLUSION:**

Attention and control of anxiety and comfort in burn patients are one of the essential elements of their care. It can be suggested that self-management training can reduce anxiety and increase burn patients’ comfort.

## INTRODUCTION

More than 95% of burns occur in developing and underdeveloped countries and affect all aspects of the life. Burning is one of the incidents which has dedicated 5 to 12% of trauma and incidents in the world^1^ and causes mortality, disability, pain, physical problems, changes in the mental image of the body,^2^ and economic and disability problems.^3^ According to the American Burn Association data, 45000 patients are hospitalized annually due to burn injuries and receive burn treatments, and about 25000 patients are admitted to specialized burn care centers.^4^

Comprehensive information about burn epidemiology is not available in Iran, and only studies in some parts of the country indicate that burns in youth, women, and people with low educational level has been higher through flames and hot liquids, and mortality rates had been changing from 27.9 to 34.4%.^5^ Based on studies undertaken in Iran, incidents, poisonings, suicide and fire have the highest mortality after circulatory diseases, and burn injury has dedicated 18% of deaths to itself, and is the second most common cause of mortality after a motor vehicle accident.^6^

Nowadays, skin grafting surgery is widely used to repair skin injuries. Skin grafting surgery is a valuable way of healing and treatment of wounds and can be considered as a process of life-saving or enhancing the life quality.^7^ Attention and control of anxiety in burn patients is one of the essential elements of patient’s care, because patients’ anxiety results in reduction of participation in therapeutic programs.^8^ Therefore, it seems that patients need to be trained about how to treat the wounds to reduce their anxiety and increase their acceptance of treatment. One of the most important aspects of nursing skills in this area is to train the patient about the care and treatments that the patient receives.^9^

Most patients report comfort feeling as one of their most important concerns after surgery.^10^ Comfort is a basic needs of human at all stages of life and providing it for patients is one of the primary responsibilities of nurses and one of their greatest challenges.^11^^,^^12^ So it is important to identify concepts of comfort and make convenience for the patients,^11^ so that whenever patients’ comfort is provided or promoted, patients show health behaviors easily and cope with stressful conditions of the disease.^13^

According to Kolb’s theory, comfort is an experience which is felt after meeting each of the palliative needs, comfort, the transcendence of the four areas including physical, psychological-mental, socio-cultural and environmental.^14^ Scars and deformities caused by burn can often lead to disorder in the mental image of self, significant weakening or losing physical function and are significantly associated with anxiety and depression.^2^^,^^15^ Nurses with a self-management approach encourage people to engage in activities that promote health, and control and treat the disease symptoms. 

Implementing the short-term training course by nurses is also effective in improving body image in the patients with burn injuries and in adopting methods that affect interpersonal functioning, emotions and relationships, and adherence of treatment regimens.^16^ Therefore, self-management training enables the patient to be actively involved in self-care and to increase the patient’s responsibility for controlling symptoms and complications. In addition, it helps the patient to maintain his/her independence and increases the performance. Researchers’ have shown that training patients before surgery and invasive procedures can reduce their anxiety.^17^ Therefore, this study was done to determine the effect of self-management training on anxiety and comfort in candidate burn patients for skin grafting.

## MATERIALS AND METHODS

This study was a clinical trial study with control group, in which the effect of self-management training on anxiety and comfort of candidate burn patients for skin grafting surgery was evaluated. The study population consisted of all candidate patients for skin grafting surgery who had been admitted to the surgical wards of men and women of Shahid Motahari Medical Center affiliated to Iran University of Medical Sciences, Tehran, Iran and had no previous skin grafting history and they were undergoing skin grafting surgery for the first time.

The study samples consisted of 80 subjects (40 in the case group and 40 in the control group). The sampling method was continuous random sampling in order to prevent bias in appointing the samples in the case and control groups and this procedure continued every other week until the samples were completed. In one week, all patients who were candidate for skin grafting surgery were placed to the case group and in the following week, all patients who were candidate for skin grafting were regarded as the control group. 

The researcher after approving the project and receiving confirmation from the Iran University of Medical Sciences Research Ethics Committee (IR.IUMS.REC.1397.258) and receiving an introduction letter from the university and presenting it to the authorities to begin work, attended in the research environment and introduced themselves to the units under study and stated the purpose of research and obtained their consent by signing a written informed consent form. Patients were included in the study before skin grafting surgery. 

Before the intervention in both case and control groups, data collection was completed through the demographic and Spiel Berger anxiety inventory and visual comfort scale questionnaires, self-management during 4 sessions including first session of familiarity with skin structure and tasks, skin graft, types of grafts and suitable places for skin grafting. The second session was training suitable time for skin grafting, skin grafting manner, skin grafting benefits, time of wounds recovery, and risks after skin grafting. 

The third session was reminder of the past issues and starting to train wound dressing, moving after skin grafting and issues which were related to care of skin grafting. The fourth session was previous review, training place of skin grafting and necessary cares about it. At the end of each session, a reception was held. In the fourth session, the questions and answers were undertaken from what they have learned to use. All sessions were held with the presence of the researcher and each session lasted 30-45 minutes in the 3-5 people groups. 

Then, during the four weeks after the intervention, the test group was given the opportunity to apply the training and again the questionnaires were completed at the end of four weeks in both groups. Both the case and control groups received routine training and a booklet was also delivered to the control group at the end of the study in order to observe ethics in the study, and the sampling were conducted in the training room, that had been previously coordinated with the training center office. 

The training has been provided by the researcher and the methods of lecture, discussion and question and answer were determined. Providing the lack of presence in one training session, the sample was removed. Data were analyzed by SPSS software (version 16, Chicago, IL, USA) and use of descriptive statistics including mean and standard deviation and inferential statistics including paired t, independent t, Fisher’s exact and Chi-squared tests at the significance level of α=0.05.

## RESULTS

The mean and standard deviation of age in the intervention group was 41.27±13.29 years and in the control group was 42.55±13.75 years old. Data analysis showed that there was no statistically significant difference between mean and standard deviation of two groups regarding demographic and contextual characteristics (Table 1). Results from the comparison of anxiety in the intervention and control groups before and one month after the intervention showed that the trait anxiety scores (*p*<0.001) and state anxiety scores (*p*<0.001) had significance difference after the intervention compared to the past state. It means that the scores after the intervention have been significantly decreased compared to previous state.

**Table 1:** Demographic characteristics of participants

**Variable**		**Intervention** **No. (%)**		**Control** **No. (%)**	***p*** ** value**
Sex	Women	17 (42.5)		17 (42.5)	
	Men	23 (57.5)		23 (57.5)	
Marital status	Single	10 (25)		8 (20)	0.340
	Married	26 (65)		31 (77.5)	
	Spouse who died and divorced	4 (10)		1 (2.5)	
Economic situation	Poor	19 (47.5)		16 (40)	0.41
	Medium	21 (52.5)		22 (55)	
	Good	0		2 (5)	
Job	Housewife	11 (27.5)		13 (32.5)	0.22
	Employee	4 (10)		2 (5)	
	Manual worker	8 (20)		9 (22.5)	
	Free	10 (25)		8 (20)	
	Unemployed	6 (15)		2 (5)	
	Retired	1 (2.5)		6 (15)	
Address	Tehran province	25 (62.5)		25 (62.5)	0.85
	Provincial center	1 (2.5)		2 (5)	
	City	14 (35)		12 (30)	
	Village	0		1 (2.5)	
Insurance status	Yes	30 (75)		31 (77.5)	0.79
	No	10 (25 )		9 (22.5)	
Type of burn	Electrical	4 (10)	1 (2.5)		0.44
	Boiling water	9 (22.5)	12 (30)		
	Fire	15 (32.5)	18 (45)		
	Other	12 (30)	9 (22.5)		

Independent t-test results demonstrated that there was no significance difference before the intervention between the intervention and control group regarding the trait anxiety scores (*p*=0.11), but in state anxiety, this difference was significant (*p*=0.006). Findings of analysis of covariance also illustrated a significance difference after the intervention between the intervention and control group (*p*<0.001, Table 2). The results of Chi-square test depicted that there was a significance difference before and after the intervention regarding the frequency of comfort levels between the control and intervention group, respectively (*p*=0.02, *p*=0.030). 

**Table 2:** Comparison of anxiety in case and control groups before and one month after intervention

**Group/Time** **Anxiety**	**Before**		**After**		**Changes before and after intervention**	
	**Case**	**Control **	**Case**	**Control **	**Case**	**Control **
Hidden anxiety (20-80)						
Mean±SD	51.50±13.27	47.26±10.13	38.88±7.18	48.80±8.65	-12.62±8.86	4.89±1.54
Domain	29-76	22-75	26-55	30-71	-(39)-6	(-8)- 8
Independent T-test	t=0.60, df=78, *p*=0.11		t=-5.57, df=78, *p*<0.001		t=-8.84, df=78, *p*<0.001	
Manifest anxiety (20-80)						
Mean±SD	56.08±12.01	49.03±10.22	38.15±7.04	48.43±8.38	-17.92±8.09	-0.6±6.74
Domain	30-75	22-68	23-52	29-71	(-5)- (-44 )	25- (-10)
Results	t=2.82, df=78, *p*=0.006*		F=16.67, *p*<0.001**		t=-10.39, df=78, *p*<*0.001	

*t-test, **Analysis of covariance

It means that before the intervention, most of the patients’ comfort levels in the intervention group were in a very upset level (40%), while in the control group, they were in relatively comfort level (27.5%). After the intervention, the highest frequency was reported in the intervention group at a relatively comfort level (40%) and after that, at a comfort level (32.5%); whereas in the control group, most of patients had a relatively comfort level (50%), and after that, it was in relatively upset level (25%) (Table 3).

**Table 3:** Comparison of comfort in case and control groups before and one month after intervention

**Group/Time** **Comfort**	**Before**				**After**			
	**Case**		**Control **		**Case**		**Control **	
	**No.**	**Percent**	**No.**	**Percent**	**No.**	**Percent**	**No.**	**Percent**
Very sad	16	40	3	7.5	0	0	1	2.5
Upset	7	17.5	10	25	1	2.5	5	12.5
Relatively upset	8	20	10	25	7	17.5	10	25
Relatively comfortable	7	17.5	11	27.5	16	40	20	50
Comfortable	1	2.5	5	12.5	13	32.5	4	10
Very comfortable	1	2.5	1	2.5	3	7.5	0	0
Total	40	100	40	100	40	100	40	100
Chi-square test	*x* ^2^=13.20, df=5, *p*=0.02				*x* ^2^=12.40, df=5, *p*=0.030			

## DISCUSSION

Our findings revealed that self-management training to candidate burn patients of skin grafting reduced the anxiety and increased the comfort. In our study, the level of comfort and the mean of trait and state anxiety scores and after implementing self-management training program had significance difference between the control and case group. Therefore, it can be said that self-management training increases the level of comfort and reduces the anxiety level of burn patients who were candidate for skin grafting. It can be concluded that self-management training to patients can be a suitable context for promoting self-management in candidate burn patients for skin grafting and thereby, increasing their comfort level and decreasing anxiety level, so a planned program should be considered for this regard. 

Mohammadi *et al.* in their study showed that group training was effective in reducing anxiety of the patients.^18^ Jasemi *et al.* in their study demonstrated that in the case group, the level of anxiety and educational needs after face-to-face training had significance reduction compared to admission time.^19^ Saki *et al.* in their study illustrated that two face-to-face and electronic training methods were effective in reducing patients’ anxiety.^20^ Due to the high percentage of comfort levels in the control and intervention group before and after the intervention, the level of comfort in the intervention group has been increased more than the control group. 

Momeni *et al.* in their study reported that training of patient and family could significantly improve comfort among hemodialysis patients. On the other hand, by reducing anxiety in these patients, the medical costs of these patients reduced and the economic burden was less in the society.^21^ Therefore, since burn is a danger that threatens people in different forms every day, self-management training can be used as a useful, low-cost and easy way to reduce anxiety and increase comfort in burn patients. One of the limitations of the present study was that the economic issues and the costs of treatment were concerns of the patients, which had a significant effect on patients’ anxiety and could increase anxiety and the researcher could not control the patients’ medical costs.
